# Vitamin D alleviates hypothyroidism associated liver dysfunction: Histological and biochemical evidence

**DOI:** 10.1016/j.heliyon.2023.e18860

**Published:** 2023-08-02

**Authors:** Seyed Hamidreza Rastegar-Moghaddam, Mahsan Akbarian, Arezoo Rajabian, Fatemeh Alipour, Alireza Ebrahimzadeh bideskan, Mahmoud Hosseini

**Affiliations:** a Student Research Committee, Mashhad University of Medical Sciences, Mashhad, Iran; b Department of Anatomy and Cell Biology, Faculty of Medicine, Mashhad University of Medical Sciences, Mashhad, Iran; c Applied Biomedical Research Center, Mashhad University of Medical Sciences, Mashhad, Iran; d Department of Internal Medicine, Faculty of Medicine, Imam Reza Hospital, Mashhad University of Medical Sciences, Mashhad, Iran; e Division of Neurocognitive Sciences, Psychiatry and Behavioral Sciences Research Center, Mashhad University of Medical Sciences, Mashhad, Iran; f Department of Physiology, Faculty of Medicine, Mashhad University of Medical Sciences, Mashhad, Iran

**Keywords:** Hypothyroidism, Liver, Fibrosis, Vitamin D, Oxidative stress

## Abstract

There is a complex correlation between thyroid hormones (THs) and liver function. Hypothyroidism as a failure of the thyroid gland to produce adequate thyroid hormones to fulfill the metabolic requirements of the body, may perturb liver structure and function. Emerging evidence suggests the protective effects of vitamin D against liver damage. Herein, this study aimed to investigate the role of vitamin D in hypothyroidism-associated liver injury. Forty male Wistar rats were classified into 4 groups: control, hypothyroid (Hypo) group received 0.05% PTU, Hypo- Vitamin D groups were given 100 and 500 IU/kg vitamin D orally via gavage for 6 weeks. Serum level of liver function including alanine aminotransferase (ALT), aspartate aminotransferase (AST), and alkaline phosphatase (ALP) were measured. Malondialdehyde (MDA) level, superoxide dismutase (SOD) enzyme activity, and total thiol content were measured as oxidative stress indicators in the liver tissue. Furthermore, to estimate liver tissue fibrosis, Masson's trichrome staining was done.

Our findings showed that hypothyroidism-induced liver fibrosis was associated with increased levels of ALT, AST and ALP. Though, vitamin D administration could significantly reduce the ALT, AST and ALP in the serum and suppress the accumulation of collagen fibers. Moreover, the activity of SOD and total thiol content was notably reduced, while the MDA content was significantly increased in the PTU- induced hypothyroid rats compared to the control group. Nonetheless, treatment with vitamin D improved mentioned oxidative stress markers in the Hypo-vitamin D groups. In conclusion, vitamin D due to its potential antioxidant and anti-fibrotic properties could be effective in the decrease of hypothyroidism-associated liver injury.

## 1 Introduction

Thyroid hormones (THs) play an essential role in tissue growth and normal functioning throughout the life [1,2]. Also, THs have important roles in the regulation of energy metabolism, mitochondrial activity, and active oxygen metabolism [2]. The liver is the major site of the peripheral metabolism of THs. Liver and the thyroid has been well documented to be closely linked, with THs playing essential roles in beta-oxidation as well as cholesterol and carbohydrate metabolism [3]. Therefore, hypothyroidism is associated with unusual lipid patterns with an increase in low-density lipoprotein (LDL) levels as well as higher total cholesterol levels [4]. On the other hand, hypothyroidism is implicated in the etiology of fibrosis and is accompanied with increased production of mucopolysaccharides, resulting in interstitial fibrosis and extracellular water retention [5,6]. The progress of fibrosis is attributable to an abnormal response determined by the accumulation of extracellular matrix proteins such as collagen and fibronectin [7]. In addition, it has been suggested that hypothyroidism can be associated with oxidative stress [8]. Indeed, hypothyroidism by changing lipid panels and increasing reactive oxygen species (ROS) generation enhances tissue oxidative damage [9]. Animal studies have shown that hypothyroidism is in correlation with an increase in the levels of biochemical markers of oxidative stress and reducing the antioxidant levels in liver tissue [2,10]. Moreover, researchers have indicated that there is a positive connection between tissue oxidative damage and fibrosis [11,12]. In the fibrotic liver, damage to hepatic cells disrupts mitochondrial chain function and increases the production of free radicals as a result of lipid peroxidation, as well as activate Kupffer cells, and hepatic stellate cells (HSCs). Among liver cells, HSCs and Kupffer cells play critical roles in fibrosis. In the fibrotic liver, Kupffer cells via releasing profibrogenic factors such as transforming growth factor beta (TGF-β) trigger HSCs activation. HSCs are the predominant precursor cells of liver myofibroblasts and upon activation, by TGF-β, fibrogenic myofibroblasts up-regulate production and deposition of α-smooth muscle actin and extracellular matrix proteins, the most abundant one being type I collagen. Likewise, experimental studies have shown that hypothyroidism can induce up-regulation of collagen type 1 gene expression, causing subsequent fibrosis [[13], [14], [15], [16]].

There is general agreement that antioxidants protect against oxidative damage by preventing the formation of free radicals [17]. Evidence shows that vitamin D, a fat-soluble secosteroid compound with pro-hormone activities, also possesses antioxidant properties by increasing the formation of glutathione (GSH) [18]. In light of this, studies have indicated that vitamin D has a potential to attenuate oxidative damage by suppressing inducible nitric oxide synthase (iNOS) expression, one of the major factors of oxidative damage. It has been reported that vitamin D can prevent lipid peroxidation in the cell membrane [[19], [20], [21]]. Vitamin D also attenuated oxidative stress in the brain of hypothyroid rats and improved neurogenesis and learning and memory [21,22]. Furthermore, it has been suggested that vitamin D plays a therapeutic role in maintaining cellular redox balance, enhancing the antioxidant pathway genes, and stimulating the expression of the potent anti-inflammatory cytokines [23]. It was also reported that vitamin D attenuated liver fibrosis, altered lipogenesis, beta-oxidation, and attenuated liver inflammation in an animal model of fructose-rich diet in mice [24]. The biological impacts of vitamin D are mediated by vitamin D receptors (VDR), a member of the transcription factor superfamily of nuclear receptors, found in most tissues, such as liver [25]. Interestingly, it has been shown that vitamin D has anti-proliferative and anti-fibrogenic effect on hepatocytes and HSCs through the transduction of vitamin D -VDR signaling via suppressing the expression of pro-fibrogenic genes and inhibiting the activity of pro-fibrotic TGF-β/suppression of the nuclear translocation of mothers against decapentaplegic homolog (SMAD) signaling pathway [26]. Moreover, it has been reported that vitamin D insufficiency and deficiency is highly prevalent in patients with some chronic liver disease-related fibrosis [27]. To the best of our knowledge, there is no published study to examine the effect of vitamin D on liver fibrosis in hypothyroidism status. Therefore, this study aimed to evaluate the potential impacts of vitamin D on liver fibrosis and oxidative stress rendered by hypothyroidism.

## 2 Material and methods

### 2.1 Chemicals

Propylthiouracil (PTU), 3-(4,5-Dimethylthiazol-2-yl)-2,5-diphenyltetrazolium (MTT) were obtained from Sigma, (St. Louis, USA). Vitamin D was purchased from the Darupakhsh Pharmaceutical Company, Iran. Hydrochloric acid (HCl), ethylenediamine tetra acetic acid disodium salt (Na2EDTA), 5,5′-Dithiobis-2-nitrobenzoic acid (DTNB), tris (hydroxymethyl) aminomethane (Trizma base), trichloroacetic acid (TCA), 2-thiobarbituric acid (TBA), dimethyl sulfoxide (DMSO) was purchased from Merck (Darmstadt, Germany). Radioimmunoassay kit (Dai source, T4-RIA - CT) and Masson's trichrome staining kit were respectively used for serum thyroxin and fibrosis measurements.

### 2.2 Animals and experimental design

In the present study, forty male Wistar rats (20 days old, weighing 50–60 g) were purchased from Mashhad University of Medical Sciences animal center and were kept in standard conditions (22 ± 2 °C and 12-h light/dark cycle) with free access to food and water. All institutional and National guidelines for the protection and principles of working with laboratory animals (NIH Publications No. 80-23, revised 1978) were implemented and approved by the Ethical Committee at Mashhad University of Medical Sciences, Mashhad, Iran (Ethical Approved Number: IR. MUMS.REC.1401.196). To induce hypothyroidism, PTU was prescribed daily for six weeks in the amount of 0.05% in drinking water [21,28,29]. The animals were randomly divided into four groups (N = 10 in each group): 1) Control group: the animals received normal drinking water as well as they were gavaged with solvent of vitamin D (saline diluted Tween 80). 2) Hypothyroid (Hypo) group: the animals received 0.05% PTU in drinking water as well as they were gavaged with solvent of vitamin D. Groups 3, 4 including Hypo-Vitamin D 100 and Hypo-Vitamin D 500 groups received 0.05% PTU in their drinking water and were gavaged with 100 and 500 IU/kg/day of vitamin D, respectively. The previous studies were considered to choose the doses of vitamin D [30,31].

After 6 weeks, the animals were deeply anesthetized with ketamine-xylazine, and blood samples were taken from the heart and centrifuged at 10000×*g* for 15 min, and the serum samples were gathered and kept at - 80 °C. The liver tissues were collected and stored at −80 °C to be used for oxidative stress assessments. To evaluate liver fibrosis, the collected tissues were fixed in 10% formalin.

### 2.3 Measurement thyroxin and liver function indicators in the serum

To confirm hypothyroidism, the serum thyroxin level was detected by radioimmunoassay method (Dai source, T4-RIA-CT). Moreover, serum concentrations of alanine aminotransferase (ALT), aspartate aminotransferase (AST), and alkaline phosphatase (ALP) were measured by standard colorimetric kits (Pars Azmun, Iran). The methods provided in the kit were followed and Autoanalyzer BT 3500 (Biotecnica, Italy) was used to measurements. All measurements of ALT, AST, ALP and thyroxin were done in a Medical laboratory (Navid, Mashhad, Iran).

### 2.4 Histological method

To evaluate liver fibrosis, the collected tissues were fixed in 10% formalin for 72 h. After fixation, liver tissues were dehydrated in alcohol, cleared in xylene, and then placed in paraffin. After preparing blocks, liver tissues were cut into 5 μm-thick serial sections using a microtome. Finally, the prepared slides were stained using Masson's trichrome stain and examined with a light microscope.

### 2.5 Masson's trichrome staining

Masson's trichrome staining was carried out to assess liver fibrosis. Liver fibrosis is determined by the excessive accumulation of collagen (blue-stained as interstitial fibrosis) and an increased quantity of connective tissue around the vessels (perivascular fibrosis). At first, the slides were deparaffinized by xylene and rehydrated in declining graded ethanol (Ethanol 100%, 90%, and 70%). After being immersed in distilled water, the slides were exposed to Hematoxylin Weigert (A and B) for 10 min. Then, the slices were washed with distilled water and immersed in acid fuchsin for 1 min. After that, the phosphomolybdic-phosphotungstic acid mixture was used for distinction (15 min). Finally, the slides were placed in an aniline blue solution for 20 min and then the slides cleared, dehydrated, and mounted [32].

### 2.6 Hepatic fibrosis assessment

Image J software was applied to quantified the collagen fibers in liver. The first step in analysis was color deconvolution using Masson's trichrome stain profiler plugin. After that, the intensity of the collagen fibers was measured in deconvoluted Masson's trichrome stain image. The average staining intensities for all measured collagen fibers from 5 fields of vision were counted for each sample. In Image J, the pixel intensity values for any color range from 0 to 255, wherein 0 represents the darkest shade and 255 represents the lightest shade of the color. In order to compare the distribution of measured staining intensities, the values measured by Image J were put into following formula. The average staining intensity was turned to an optical density (OD). The intensity of the blue color (collagen fibers) indicates the amount of collagen formation [32,33].

$$Optical\;Density=\mathrm{Log}\left(\frac{\mathrm{Max}\;intensity}{Mean\;intensity}\right)$$

The max intensity in image J is 255.

### 2.7 Biochemical appraisal

The homogenates (10 %w/v) of the collected liver tissues were prepared in ice-cold phosphate buffer saline (PBS) (0.1 M, pH 7.4). The homogenates were then centrifuged at 4 °C (10000×*g*), and the supernatants were separated to assess total thiol and malondialdehyde (MDA) concentrations as well as superoxide dismutase (SOD) activity.

### 2.8 Estimation of MDA content

As a primary indicator of lipid peroxidation, MDA concentration was measured in the liver tissues. At first, a reagent including a mixture of TCA/TBA/HCl were prepared. Then, 1 ml of the tissue homogenates was added to the tubes containing 2 ml of the reagent. The tubes were then placed in a boiling water bath for 45 min. In the next step, the solution was allowed to cool and its temperature came with the ambient. Then, the solutions were centrifuged at 1000×*g* for 10 min. Finally, after removing the supernatant, the absorbance of the pink complex was read at a wavelength of 535 nm using a spectrophotometer. The MDA concentration was calculated using the previously described formula [34,35].

### 2.9 Estimation of total thiol content

DTNB reagent was used to measure the total amount of thiol groups. This reagent reacts with sulfhydryl (SH) groups and creates a yellow complex with maximum absorbance at 412 nm. To measure thiol content, 1 ml of Tris-EDTA buffer (30 mM Tris), 3 mM EDTA pH = 8.2) was added to 50 μl of the homogenous sample and the first absorbance was read at 412 nm using a spectrophotometer against Tris-EDTA buffer alone (A1). Then, 20 μl of DTNB reagent (in methanol) was added to each sample, and the second absorption of the sample was read (A2). The total amount of thiol (mM) was calculated following an equation previously described [21,36]. Finally, the results were reported as units per gram of tissue.

### 2.10 Estimation of SOD activity

SOD activity was measured based on Madesh and Balasubramanian's colorimetric method using 96-well plates [37]. This method is based on the production of superoxide through auto-oxidation of pyrogallol and inhibition of superoxide-dependent reduction of MTT to its formazan. The reaction was stopped by adding DMSO, which allows for the dissolution of the formazan and stabilizes the color formed. Briefly, 60 μl of homogenized tissue, MTT, and pyrogallol was poured into the wells and incubated for 5 min at room temperature and in the dark. The colorimetric changes were measured at 570 nm with an ELISA reader. One unit of SOD activity was determined as the quantity of enzyme that leads to 50% deterrence in the MTT reduction rate.

### 2.11 Statistical analysis

All data were analyzed using SPSS software (version 26 Chicago, IL), by one-way ANOVA followed by Tukey's post hoc comparisons test, and represented as mean ± SEM. p < 0.05 was considered statistically significant.

## 3 Results

### 3.1 Thyroxin level profile

**Fig. 1:**
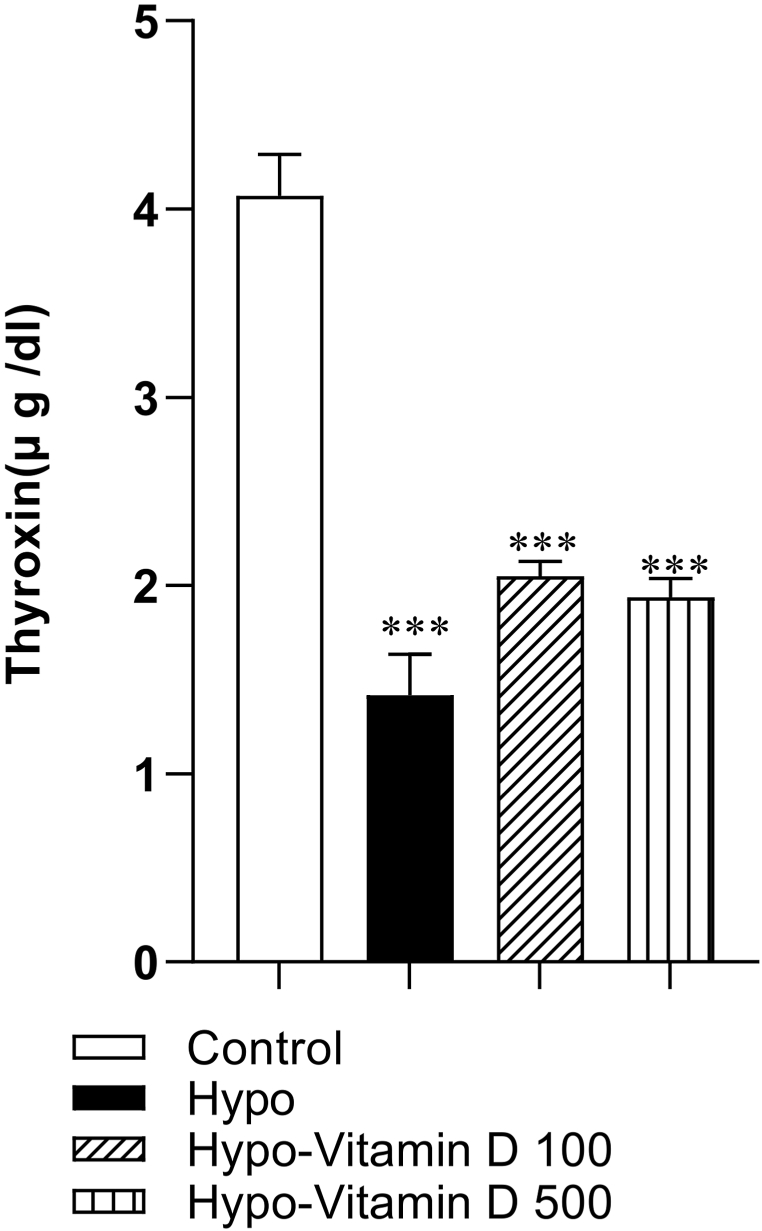
The effect of Vit D on the serum level of thyroxin. The data are presented as mean ± SEM (n = 8–10). ***p < 0.001 compared to the Control group.

To confirm hypothyroidism status, serum thyroxin level was measured. The results showed that PTU administration induced hypothyroidism. The results revealed that the serum thyroxin level was significantly lower in the Hypo, Hypo-Vitamin D100, and Hypo-Vitamin D500 groups compared to the Control group. (p < 0.001). However, no significant difference was observed in the serum level of thyroxin among the Hypo, Hypo-Vitamin D 100, and Hypo-Vitamin D 500 groups compared together. Based on the mentioned results, Vitamin D was not able to correct T4 concentration (Fig. 1).

### 3.2 Serum level of hepatic biomarkers (AST, ALT, and ALP)

**Fig. 2:**
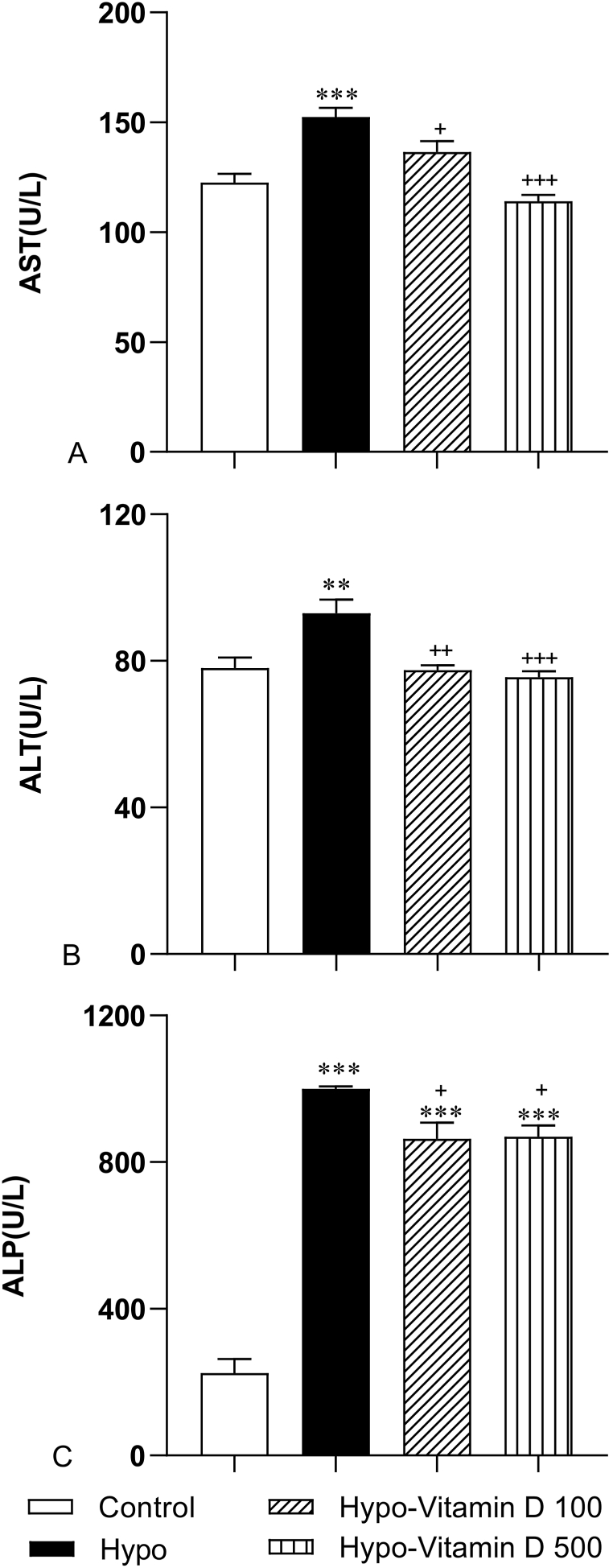
The effect of Vit D on liver function indicators including aspartate transferase (AST), alanine aminotransferase (ALT), and alkaline phosphatase (ALP). The data are presented as mean ± SEM (n = 8–10 for AST and ALT and n = 6–8 for ALP). (A) AST: *** significance against the Control group (p < 0.001),+and ^+++^ significance against the Hypo group (p < 0.05 and p < 0.001). (B) ALT: ** significance against Control group (p < 0.01), ^++^ and ^+++^ significance against Hypo group (p < 0.01 and p < 0.001). (C) ALP: *** significance against Control group (p < 0.001), *** significance against Hypo group (p < 0.001),+significance against Control group (p < 0.05).

The serum levels of AST, ALT, and ALP are representative markers that reflect the degree of liver damage. The results of this study showed that hypothyroidism caused a significant rise in the level of AST, ALT, and ALP enzymes compared to the Control group (p < 0.001, p < 0.01, and p < 0.001). This result indicates that hepatocytes were significantly damaged in the PTU- treated hypothyroid rats. In more detail, the mean of AST, ALT, and ALP of the Hypo group was markedly higher than in the groups Hypo-Vitamin D 100 (p < 0.05, p < 0.01, and p < 0.05 respectively) and Hypo-Vitamin D 500 (p < 0.001, p < 0.001, and p < 0.05 respectively) groups. These results suggest that vitamin D can normalize the levels of AST, ALT, and ALP. In terms of ALP, the groups treated with both 100 and 500 IU/kg of vitamin D had a significantly higher ALP concentration than that of the Control group (both p < 0.05) (Fig. 2).

### 3.3 Histopathological changes

**Fig. 3:**
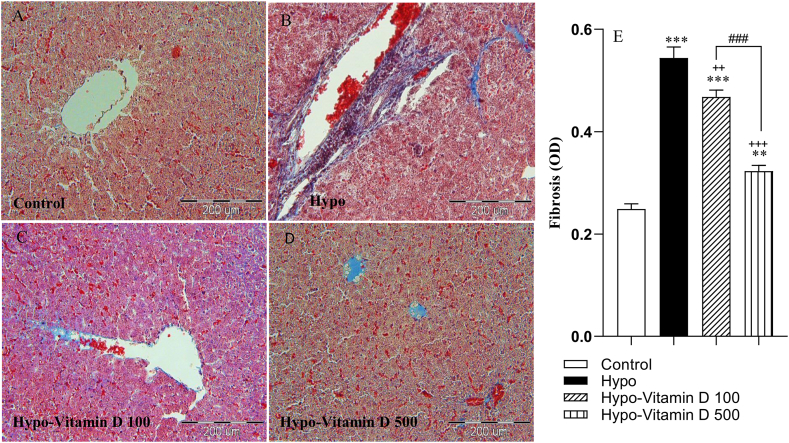
(A–D) Microscopic pictures of Masson's trichrome staining that present the distribution of collagen fibers (blue) in the background of the liver (dark purple and pink) (200x). Scale bar: 200 μm. The normal architecture of liver tissue is observed in the control group, whereas all livers of the Hypo groups had different degrees of fibrosis. (E) The graph shows the comparison of liver fibrotic areas among the experimental groups. The data are presented as mean ± SEM (n = 5). The average optical density (OD) values were observed using the Image J analysis software. ***p < 0.001 and **p < 0.01 vs control group, ^++^p<0.01 and ^+++^p<0.001 vs Hypo group, ^###^p < 0.001 vs Hypo-vitamin D 100 group.

Histologically, we employed Masson's trichrome staining to present the distribution of collagen fibers in the liver. Images were analyzed by Image J software and showed the blue area represented by the collagen fiber area in the different groups. Compared with the control group, the livers of the PTU- treated hypothyroid rats developed typical liver fibrosis, which revealed rapid increases in fibrotic tissue area (p < 0.001). Cord-like thick collagen fibers could be found in the disrupted lobular areas in the liver sections of the Hypo group. Treatment with vitamin D at both doses and especially 500 IU/kg significantly ameliorated these alterations as short bands of collagen. Measurements of the collagen area of the Hypo-Vitamin D 100 and Hypo-Vitamin D 500 groups were significantly lower than the Hypo group (p < 0.01, p < 0.001, respectively). The results also showed that fibrotic tissue areas in the three Hypo, Hypo-Vitamin D 100, and Hypo-Vitamin D 500 groups were significantly higher than those of the control group (p < 0.001, p < 0.001, and p < 0.01, respectively). Moreover, treatment with 500 IU/kg vitamin D yielded a pronounced reduction in hepatic fibrosis in comparison with 100 IU/kg vitamin D (p < 0.001) (Fig. 3A–E).

### 3.4 The results of oxidative stress indicators

#### 3.4.1 Vitamin D reduced MDA level in liver

**Fig. 4:**
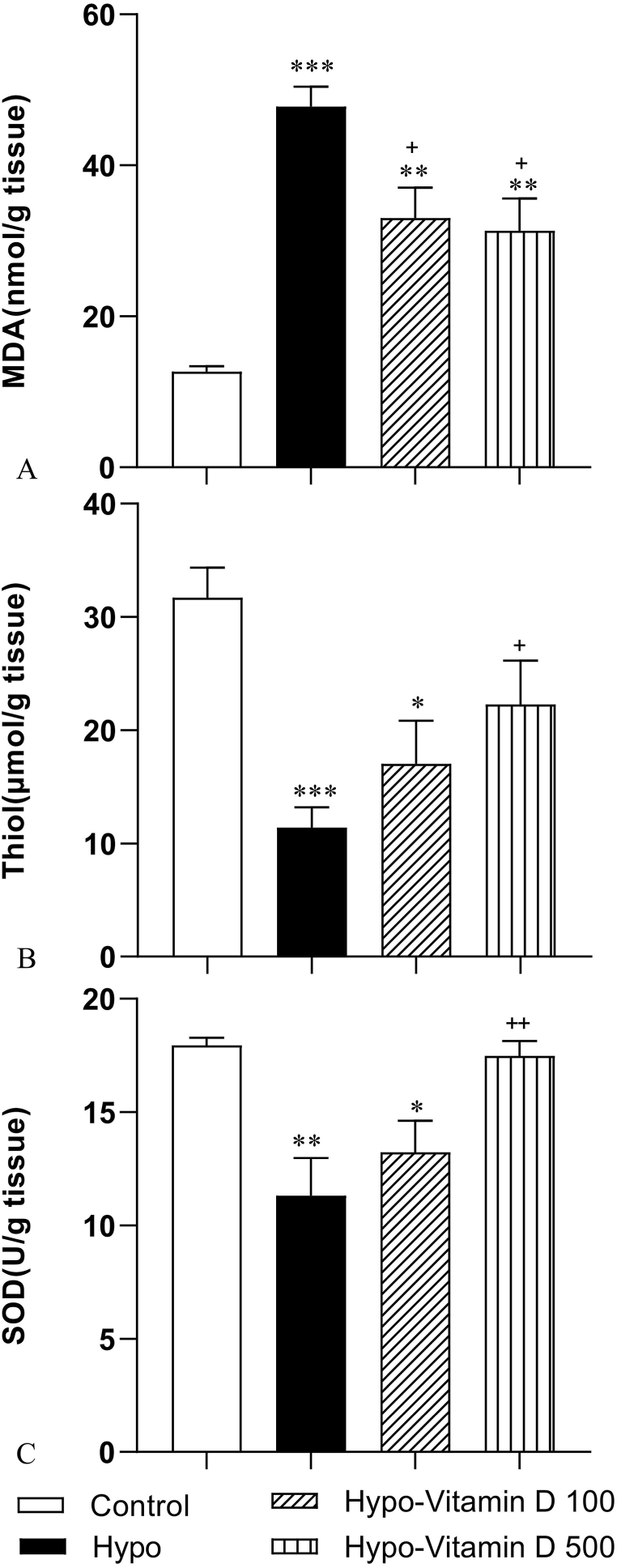
(A) Comparison of MDA level as a lipid peroxidation marker in the liver among the experimental groups. Values represent mean ± SEM (n = 10). **p < 0.001 and ***p < 0.001 compared to the control group, ^+^p<0.05 compared to the Hypo group. (B) Comparison of thiol content in the liver between the experimental groups. Values represent mean ± SEM (n = 8–10).*p < 0.05 and ***p < 0.001 compared to the control group, ^+^p<0.05 compared to the Hypo group. (C) Comparison of SOD activity between the experimental groups. (n = 10). *p < 0.05 and **p < 0.01 compared to the control group, ^++^p<0.01 compared to the Hypo group.

Fig. 4A shows compares the MDA content of the liver tissue among groups. The comparisons test indicated that the MDA level was significantly elevated in the Hypo group compared with the Control group (p < 0.001). However, vitamin D administration remarkably reduced the MDA level in both Hypo-Vitamin D 100 and Hypo-Vitamin D 500 groups compared to the Hypo group (both p < 0.05).

#### 3.4.2 Vitamin D enhanced total thiol content in liver

Fig. 4B shows the total thiol content of the liver tissue in the experimental groups. In the Hypo group, a significant decrease was observed in the level of liver total thiol in comparison with the Control group (p < 0.001). Also, this amount was lower in the group of Hypo-Vitamin D 100 compared to the control group (p < 0.05), whereas treatment with 500 IU/kg of vitamin D significantly prevented hypothyroidism-induced loss of thiols in the liver tissue (p < 0.05).

#### 3.4.3 Vitamin D improved SOD activity in liver

Fig. 4C shows that the SOD activity in the liver tissue of the experimental groups. A significant reduction in the SOD activity was observed in the Hypo (p < 0.01) and Hypo-Vitamin D 100 group (p < 0.05) compared with the Control group. However, the SOD activity in the Hypo-Vitamin D 500 group was significantly increased compared to the Hypo group (p < 0.01).

## 4 Discussion

The results of the present study indicated that hypothyroidism was accompanied by an elevated level of hepatic enzymes and had severe histological changes in hepatic tissue, which confirmed liver damage. In this investigation, PTU administration was followed by a strong decrease in serum thyroxin level therefore it seems that PTU induced an overt hypothyroidism status [[38], [39], [40]]. THs have been shown to regulate the basal metabolic rate of all cells, including hepatocytes, and thereby thyroid dysfunction may perturb liver function [41]. Moreover, thyroid dysfunction has been associated with several liver diseases, such as chronic hepatitis C, liver cirrhosis, hepatocellular carcinoma, and cholangiocarcinoma [42]. There is also evidence that hypothyroidism may adversely affect liver structure or function [41]. On the other hand, the serum activities of liver enzymes, including AST, ALT, and ALP are the markers for assessing liver function [43]. The current study showed that the hypothyroid animals had a sharp increment in the activity of these markers, which may indicate that hepatocytes were significantly damaged in hypothyroidism induced by PTU in rats. Thus, high levels of liver enzymes in serum are indicators of abnormal liver function resulting in their release into the bloodstream from damaged liver cells [44]. Consistent with our results, other studies revealed that the serum levels of AST and ALT increased in hypothyroid rats [42]. Furthermore, the histological results of the present study showed that hypothyroidism leads to liver fibrosis which was confirmed by Masson staining a convenient method for quantifying collagen fibers.

This histopathological characteristic showed that experimentally-induced hypothyroidism developed liver fibrosis, concomitant with the development of an abnormal liver function [45]. Liver fibrosis is characterized by the continuous accumulation and excessive deposition of hepatic extracellular matrix (ECM) and altered liver function [46]. Excess ECM, which is caused by an imbalance between the synthesis and degradation of ECM components, plays a key role in the pathogenesis of liver fibrosis. Fibrotic liver leads to an increased biosynthesis or decreased degradation of collagens, the major component of ECM(47). Several mechanisms have been proposed that explain the correlation between thyroid function and fibrotic diseases, including changes in collagen gene expression, collagen deposition, fibro-genic cytokines activity, and unbalanced redox reactions [47]. Based on the experimental studies, thyro-mimetic compounds which are selective for THs have shown anti-fibrotic properties, whereas genetically ablated THs receptors lead to the development of fibrosis [5,48]. Similarly, according to some previous studies, fibrosis in the liver, heart, and lungs can be promoted by hypothyroidism and can be reversed by administration of THs [5,49]. Furthermore, hypothyroidism can cause fibrosis via up-regulating the gene expression of collagen type I whereas THs treatment can reverse these changes [50]. Metalloproteinase as an essential part of regulating the degradation of ECM has been implicated in the pathogenesis of liver fibrosis. THs are capable of enhancing the matrix metalloproteinase activity, further resulting in a collagen breakdown. In addition, TGF-β, a potent pro-fibrogenic cytokine, is responsible for ECM synthesis including collagen I as well as III and enhances the expression of matrix metalloproteinase [26,51]. It was found that in hypothyroid patients, TGF-β promoted collagen transcription and resulting in liver fibrosis. On the other hand, THs can antagonize the progression of fibrosis by inhibiting the TGFβ/SMAD-dependent transcriptional activation that capable of inducing fibro-genesis [52].

Our results also revealed that the elevated level of lipid peroxidation was associated with reduction in the total thiol content, and SOD activity in the hypothyroid group, implying the occurrence of oxidative stress in the liver. Thus, it is suggested that the hypothyroid-induced hepatic injury was mediated partly through the oxidative stress condition. These findings are in agreement with the previous studies that showed oxidative stress in liver is probably due to the over-production of ROS and disturbance in the antioxidant defense system following hypothyroidism [53]. Therefore, it seems that hypothyroidism may increase ROS level by decreasing SOD activity and total thiol content and as a result, ROS overload can disrupt lipid membrane architecture of the hepatocytes and loss of hepatocyte function [54,55]. THs have been documented to increase the basal metabolic rate and stimulate oxygen consumption in metabolically active cells by promoting the action of Na^+^/K^+^ ATPases, increasing the mitochondrial number and mitochondrial gene expression [56]. Hypothyroidism has been associated with mitochondrial dysfunction and increased production of ROS, which contribute to cell apoptosis and fibrosis. This can be restored by THs, which improve mitochondrial function and attenuate oxidative stress [5].

In the present study, we also investigated the possible protective effect of vitamin D against hypothyroidism-induced hepatotoxicity. To the best of our knowledge, there was no published study to examine the effect of vitamin D on liver fibrosis and oxidative damage in hypothyroidism. Our primary assumption was whether there is a minimum therapeutic dose of vitamin D to improve liver fibrosis or not. In addition, the previous studies have determined that administering 100 or 500 IU/Kg of vitamin D could positively impact on liver damage [30,31]. In light of this, we also included this dose in the study. Our result demonstrated that the administration of 100 and 500 IU/kg vitamin D prevented hypothyroidism-induced liver injury. However, the administration of 500 IU/kg vitamin D was more effective in preventing hypothyroidism-induced liver fibrosis and oxidative damage, suggesting a dose-response relationship. Hypothyroid animals that received vitamin D had a significant decrease in serum level of liver enzymes along with improved pathological features in the liver tissues compared to the hypothyroid group. In addition, we found that vitamin D alleviated hypothyroid-induced oxidative stress; therefore, it is suggested that the hepato-protective effect of vitamin D against hypothyroidism-induced hepatotoxicity is partly mediated through its antioxidant features [26].

Interestingly, previous experimental studies have demonstrated that vitamin D has anti-fibrotic properties on the liver. The results of a meta-analysis suggested the beneficial effects of vitamin D on the progression of liver fibrosis [57] and exerts its function by blocking the activation of the pro-fibrotic TGFβ/SMAD pathway [26,27]. It is generally recognized that VDR is expressed in hepatocytes and the hepatic non-parenchymal cells, including HSCs and biliary epithelial cells [58]. Further, it is hypothesized that the liver may initially respond to vitamin D through its non-parenchymal cells. Nonetheless, it is well-documented that HSCs activation is responsible for extracellular matrix deposition during fibrogenesis via transforming into myofibroblasts. Consequently, it may be argued that vitamin D has a crucial role in the control of the fibrotic process by suppressing collagen production in stromal HSCs [59,60]. In our results, hypothyroidism was accompanied with a significant reduction in serum thyroxin levels in the PTU-administered groups compared to the control group. However, treatment with vitamin D has not improved the thyroxin concentration. It seems that vitamin D ameliorated liver fibrosis, as well as reduced tissue oxidative damage without affecting thyroxin serum levels. Indeed, Vitamin D did not inhibit the impact of PTU on the thyroid gland. Previous researches have shown that hypothyroidism is associated with an oxidative stress status [2,10]. Moreover, a positive relation between tissue oxidative damage and fibrosis has been reported [11,12]. In a previous study, we showed that vitamin C as a well-known anti-oxidant improved liver and renal functions in hypothyroid rats [61]. The results of the current study indicated that the vitamin D treatment at dose of 500 IU/Kg substantially reduced MDA and improved thiol content and SOD activity in the liver. Therefore, as a mechanism, it can be suggested that the beneficial effects of vitamin D on liver fibrosis is partially associated with the inhibition of oxidative damage. However, more future investigations are needed to warrant further confirming our findings and determine the exact mechanism (s) of the effects of vitamin D on hypothyroidism associated liver fibrosis. Taken together, it should be emphasized that there were significant associations between hypothyroidism and liver fibrosis. From the above-mentioned reports, vitamin D supplementation might be highlighted as a new alternative therapeutic option against fibrosis progression [26,62]. A limitation of the present study was that here only male rats were used. Considering the existence of hormonal changes in the menstrual cycles of females, male animals were used in the current research. Based on the sex differences in the manifesting of most diseases, a similar study with female animals is advocated, especially since the higher prevalence of hypothyroidism in females than the males.

The anti-thyroid drug PTU has been reported to have some side effects including hepatotoxicity [63,64]. These side effects might be protected by the anti-oxidants and corticosteroids including curcumin, prednisolone and glutathione [64,65]. Considering these evidence it seems that vitamin D has a protective impact on side effects of anti-thyroid drugs including PTU however it needs to be more investigated.

## 5 Conclusions

Our results suggest that thyroid dysfunction is associated with liver damage characterized by abnormal liver function tests as well as liver fibrosis and redox homeostasis. On the other hand, vitamin D supplementation could ameliorate oxidative injury along with changes in liver structure and function in the hypothyroid animals. Vitamin D, with its unique protective effects, has the potential to be repurposed as a therapeutic agent for hepatic diseases associated with fibrosis. Nevertheless, more research still needs to be focused on the regulatory role of vitamin D in inhibiting liver fibro-genesis and to assess the safety and efficiency of this supplementation as a widely available and relatively inexpensive treatment for liver fibrotic conditions.

## Author contribution statement

Seyed Hamidreza Rastegar-Moghaddam, Mahsan Akbarian: Performed the experiments; Wrote the paper.

Arezoo Rajabian: Contributed reagents, materials, analysis tools or data.

Fatemeh Alipour: Contributed reagents, materials, analysis tools or data; Wrote the paper.

Alireza Ebrahimzadeh Bideskan: Conceived and designed the experiments; Analyzed and interpreted the data.

Mahmoud Hosseini: Conceived and designed the experiments; Analyzed and interpreted the data; Wrote the paper.

## Funding statement

This work was supported by the Vice Chancellor for Research, at 10.13039/501100004748Mashhad University of Medical Sciences, Mashhad, Iran {4010485}.

## Data availability statement

Data will be made available on request.

## Additional information

No additional information is available for this paper.

## Declaration of competing interest

The authors declare that they have no known competing financial interests or personal relationships that could have appeared to influence the work reported in this paper.
